# Chitin-glucan supplementation improved postprandial metabolism and altered gut microbiota in subjects at cardiometabolic risk in a randomized trial

**DOI:** 10.1038/s41598-022-12920-z

**Published:** 2022-05-25

**Authors:** Harimalala Ranaivo, Zhengxiao Zhang, Maud Alligier, Laurie Van Den Berghe, Monique Sothier, Stéphanie Lambert-Porcheron, Nathalie Feugier, Charlotte Cuerq, Christelle Machon, Audrey M. Neyrinck, Benjamin Seethaler, Julie Rodriguez, Martin Roumain, Giulio G. Muccioli, Véronique Maquet, Martine Laville, Stephan C. Bischoff, Jens Walter, Nathalie M. Delzenne, Julie-Anne Nazare

**Affiliations:** 1 Centre de Recherche en Nutrition Humaine Rhône-Alpes, Hospices Civils de Lyon, CENS, FCRIN/FORCE Network, Pierre-Bénite, France; 2 Univ-Lyon, CarMeN Laboratory, INSERM, INRAE, Université Claude Bernard Lyon-1, 69600 Oullins, France; 3 College of Food and Biological Engineering, Jimei University, Xiamen, Fujian China; 4 Service de Biochimie et Biologie Moléculaire, Unité Médicale Dyslipidémies et Dysfonctions Nutritionnelles et Digestives, Hospices Civils de Lyon, Pierre-Bénite, France; 5 Hospices Civils de Lyon, Service de Biochimie, Centre de Biologie Sud, Hôpital Lyon Sud, Pierre-Bénite, France; 6 Metabolism and Nutrition Research Group, Louvain Drug Research Institute, UCLouvain, Université Catholique de Louvain, Ottignies-Louvain-la-Neuve, Belgium; 7 Institute of Nutritional Medicine, University of Hohenheim, Stuttgart, Germany; 8 Bioanalysis and Pharmacology of Bioactive Lipids Research Group, Louvain Drug Research Institute, UCLouvain, Université Catholique de Louvain, Brussels, Belgium; 9 KitoZyme, Parc Industriel des Hauts-Sart, Zone 2, Rue de Milmort 680, 4040 Herstal, Belgium; 10 Department of Medicine, and School of Microbiology, APC Microbiome Ireland, University College Cork, Cork, Ireland

**Keywords:** Metabolic diseases, Biomarkers, Microbiome, Dyslipidaemias, Metabolic syndrome, Nutrition, Risk factors

## Abstract

Chitin-glucan (CG), an insoluble dietary fiber, has been shown to improve cardiometabolic disorders associated with obesity in mice. Its effects in healthy subjects has recently been studied, revealing its interaction with the gut microbiota. In this double-blind, randomized, cross-over, twice 3-week exploratory study, we investigated the impacts of CG on the cardiometabolic profile and gut microbiota composition and functions in 15 subjects at cardiometabolic risk. They consumed as a supplement 4.5 g of CG daily or maltodextrin as control. Before and after interventions, fasting and postprandial metabolic parameters and exhaled gases (hydrogen [H_2_] and methane [CH_4_]) were evaluated. Gut microbiota composition (16S rRNA gene sequencing analysis), fecal concentrations of bile acids, long- and short-chain fatty acids (LCFA, SCFA), zonulin, calprotectin and lipopolysaccharide binding protein (LBP) were analyzed. Compared to control, CG supplementation increased exhaled H_2_ following an enriched-fiber breakfast ingestion and decreased postprandial glycemia and triglyceridemia response to a standardized test meal challenge served at lunch. Of note, the decrease in postprandial glycemia was only observed in subjects with higher exhaled H_2_, assessed upon lactulose breath test performed at inclusion. CG decreased a family belonging to Actinobacteria phylum and increased 3 bacterial taxa: *Erysipelotrichaceae* UCG.003, *Ruminococcaceae* UCG.005 and *Eubacterium ventriosum group*. Fecal metabolites, inflammatory and intestinal permeability markers did not differ between groups. In conclusion, we showed that CG supplementation modified the gut microbiota composition and improved postprandial glycemic response, an early determinant of cardiometabolic risk. Our results also suggest breath H_2_ production as a non-invasive parameter of interest for predicting the effectiveness of dietary fiber intervention.

## Introduction

The beneficial effects of dietary fibers (DF) are now well recognized and the current recommended daily intake is 25–30 g/day^1^.When assessing DF contribution to health, DF interaction with gut microbiota is also widely reported as of interest. DF may actually affect gut microbiota composition and function and induce the production of gut-derived metabolites such as short-chain fatty acids (SCFA), which in turn can improve glucose and lipid parameters^2^. DF can have different origins and can be classified according to their physicochemical properties (including solubility, viscosity or fermentability)^3^. These parameters are used to classify DF which are thus subdivided into resistant starch (RS), non-starch polysaccharides (NSPs) and resistant oligosaccharides (ROs)^4,5^. DF also have different physiological effects which are not always associated with their classification, particularly in terms of their solubility^5,6^. Actually, solubility is often correlated with fermentability so that soluble DF are fermented more quickly than insoluble DF, with the exception of RS which are highly fermentable although the majority of them are insoluble DF^6,7^. Of note, the health effects and the underlying mechanisms of action of insoluble DF remain unclear. In this context, the project FiberTAG (Joint Programming Initiative “A Healthy Diet for a Healthy Life” 2017–2020 https://www.fibertag.eu/) aimed at establishing a set of biomarkers of gut barrier function and bacterial co-metabolites beyond SCFA, such as long-chain fatty acids (LCFA), bile acids (BA), intestinal permeability and endotoxemia, which link DF intake and gut-microbiota related health effects. Moreover, we also focused on noninvasive biomarkers such as exhaled gases^8^. In particular, lactulose hydrogen (H_2_) breath test is primarily used as a marker for small intestinal bacterial overgrowth (SIBO). Since several reports compared the fermentation of DF using the measurement of end-expiratory H_2_ concentrations and suggested that they depend on the type of DF^9^, exhaled H_2_ during lactulose H_2_ breath test could be of interest as it would allow to assess the fate of DF and its cardiometabolic effects.

Here, we were particularly interested in chitin-glucan (CG), a novel insoluble DF considered as safe food ingredient by the European Food Safety Authority^10^. It is the major component of the cell walls of the fungi *Aspergillus niger* and is mainly composed of a branched β-1, 3/1, 6 glucan that is linked to chitin via a β-1, 4 linkage. Preclinical studies in high fat diet (HFD) rodent models showed potential beneficial effects of CG supplementation, namely on gut microbiota composition and function but also on the cardiometabolic profile^11,12^. The first attempt to study the effect of this CG on gut microbiota in humans was carried out as part of an in vitro model using a Simulator of the Human Intestinal Microbial Ecosystem (SHIME)^13^. This study showed that CG was fermented in the colon and induced the production of gut-derived metabolites such as short chain fatty acids (SCFA). Consistently, CG-derived effects on gut microbiota and bacterial metabolites in humans have been studied recently, revealing the interaction of CG with the gut microbiota in healthy subjects^14^. Together, these results suggest that it would be of interest to study the effects of CG in subjects at cardiometabolic risk who may benefit more distinctly than healthy individuals from such intervention.

In this double-blind, randomized, cross-over exploratory study, we aimed at assessing whether a 3-week daily supplementation of 4.5 g of CG had different effects from maltodextrin (as control) on gut microbiota composition and functions as well as cardiometabolic profile (including postprandial phenotyping) in subjects at cardiometabolic risk (CMR) by assessing a comprehensive set of biomarkers. We also investigated the relevance of targeted biomarkers to identify responders to the fiber intervention, allowing us to further characterize those participants who were able to metabolize CG.

## Results

### Participant characteristics, compliance, and dietary intake

**Figure 1:**
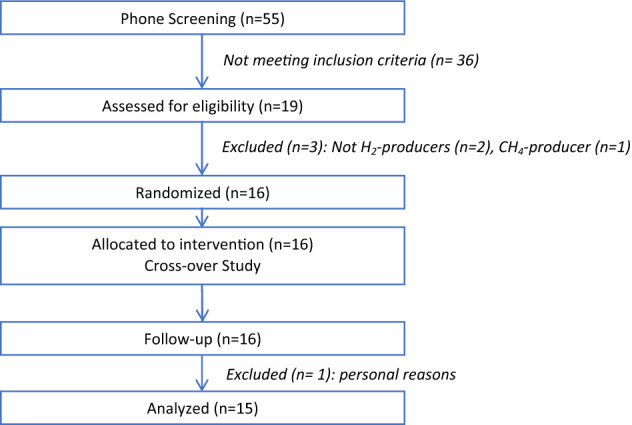
CONSORT flow diagram of participants.

**Figure 2:**
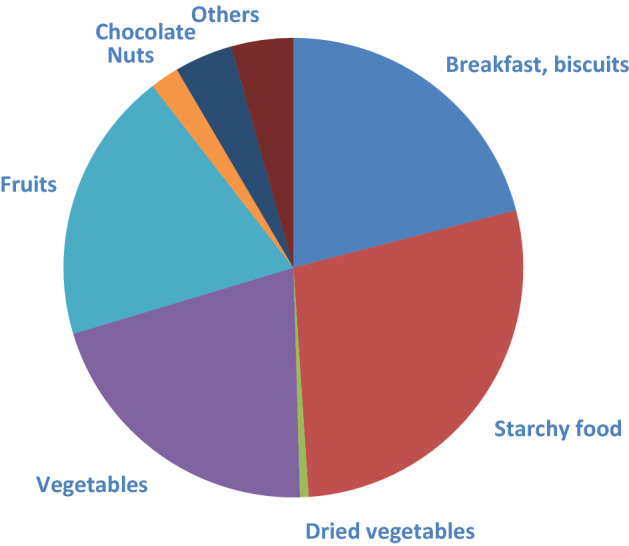
Origin of consumed dietary fibers at baseline (g/d).

Sixteen subjects with CMR profile were included and randomized: 15 completed the cross-over study (9 men, 6 women) and were analyzed (Fig. 1). They were overweight/obese, showed an elevated waist circumference (abdominal obesity), and had neither fasting hyperglycemia, diabetes, elevated high-sensitivity C-reactive protein levels, nor hypertension (Table 1). 90% were dyslipidemic (abnormal fasting concentration of triglycerides and/or total cholesterol and/or high- and low density lipoprotein cholesterol (HDL and LDL cholesterol)). The mean daily fiber intake was 19 ± 4 g/day and the mean increase over the baseline of exhaled hydrogen concentration following the lactulose breath test was 84 ± 44 ppm. The compliance to study product consumption was high (96%) and dietary records showed that subjects followed the instructions they were given. No modification of their physical activity has been reported and they actually did not modify their energy, macronutrient and dietary fiber intakes – excluding CG – throughout the study (Table 2). At baseline, consumed fiber-rich food items were mainly from starchy food, vegetables and fruits (Fig. 2) and this was not modified throughout the study (data not shown).

**Table 1:** Subjects’ anthropometric and metabolic characteristics at baseline (n = 15).

	Mean ± SD
**Anthropometry**	
Age (years)	44 ± 10
Weight (kg)	83 ± 13
Height (m)	1.7 ± 0.1
BMI (kg/m^2^)	28 ± 2
**Waist circumference (cm)**	
Male	104 ± 8
Female	93 ± 6
**Hip circumference (cm)**	
Male	107 ± 6
Female	107 ± 4
**Blood pressure (mmHg)**	
Systolic pressure	123 ± 11
Diastolic pressure	75 ± 11
**Blood biomarkers**	
Glucose (mM)	5.2 ± 0.41
HbA1c (%)	5.41 ± 0.26
TG (mM)	1.19 ± 0.42
TC (mM)	5.52 ± 0.86
HDL-C(mM)	1.24 ± 0.30
LDL-C (mM)	3.73 ± 0.81
hs-CRP (mg/L)	3.15 ± 2.88
AST (UI/L)	28 ± 10
ALT (UI/L)	35 ± 28

Data are expressed as mean ± SD. BMI, body mass index; HbA1c, glycated hemoglobin; TG, triglyceride; TC, total cholesterol, HDL-C, high density lipoprotein cholesterol, LDL-C, low density lipoprotein cholesterol; hs-CRP, high sensitivity C reactive protein; AST, Aspartate Aminotransferase; ALT, Alanine Aminotransferase.

**Table 2:** Daily dietary intake throughout the study (n = 15).

	CG		CTL		Time
	Before	After	Before	After	*p* value
Energy (kcal)	2174 ± 414	1938 ± 323	1928 ± 295	1992 ± 257	ns
Total carbohydrate (g)	233.5 ± 43.1	212.6 ± 30.1	214.0 ± 38.6	215.7 ± 43.8	ns
Protein (g)	83.9 ± 18.9	73.6 ± 17.6	74.2 ± 12.0	82.9 ± 16.9	ns
Fat (g)	92.4 ± 26.0	80.9 ± 20.0	79.9 ± 18.2	81.2 ± 14.1	ns
Saturated fat (g)	38.8 ± 11.2	34.5 ± 10.3	34.1 ± 8.7	34.3 ± 8.2	ns
Alcohol (g)	4.5 ± 6.9	3.8 ± 9.3	2.2 ± 4.2	3.7 ± 7.1	ns
Fibers (g)	18.0 ± 5.1	17 ± 3.8	16.7 ± 4.6	17.2 ± 6.1	ns

Data are expressed as mean ± SD. CG, chitin glucan; CTL, control. A mixed linear model with treatment, time, period and sequence as fixed variables and subjects as random effect has been performed. None of the *p* values associated to time is significant.

### CG increased exhaled H_2_ and improved postprandial glycemic and lipemic profiles

**Figure 3:**
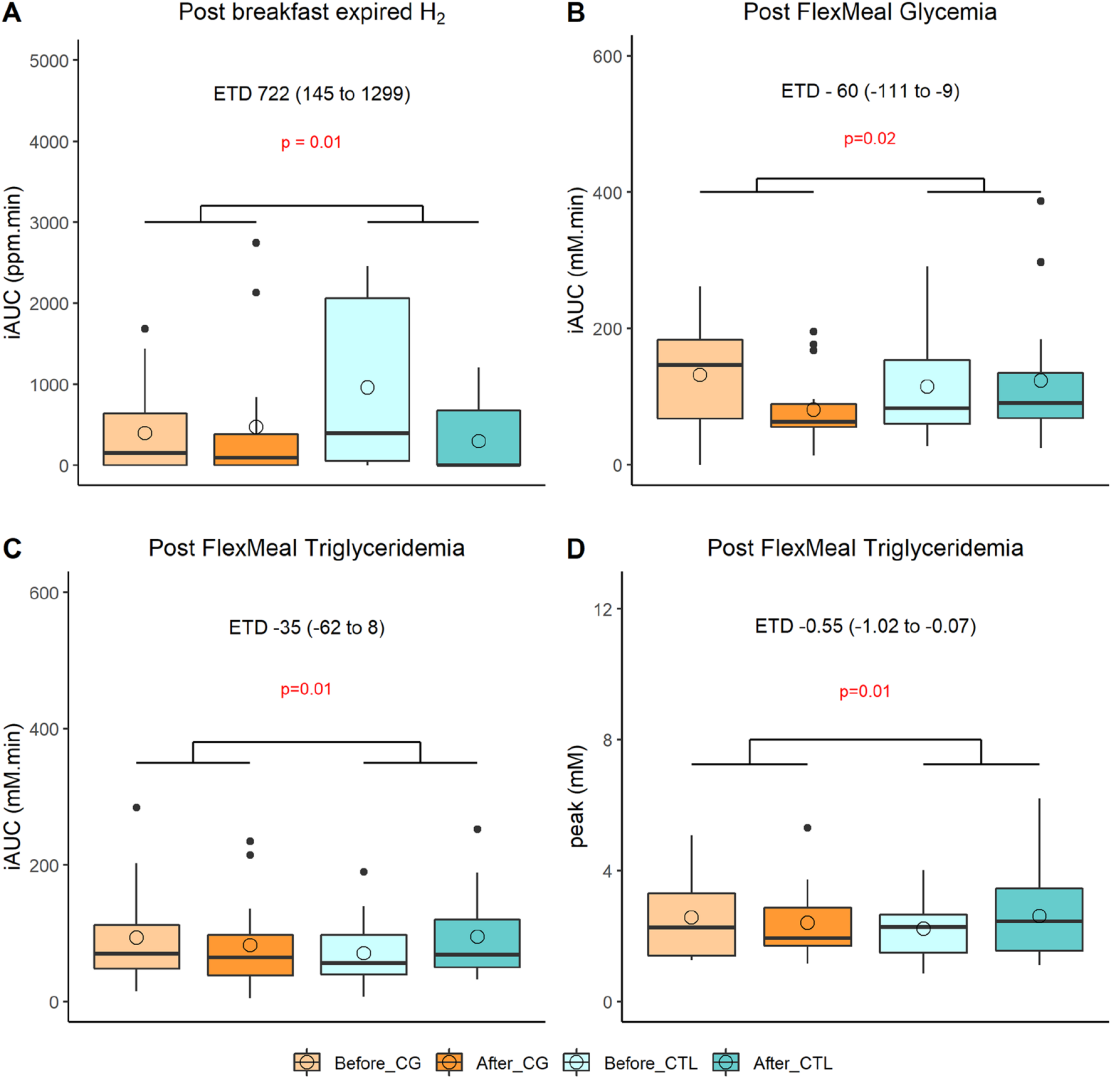
Impacted postprandial metabolic parameters (n = 15). (**A**) iAUC of postprandial exhaled H_2_ following the ingestion of a fiber-enriched breakfast, iAUC of postprandial glycaemia (**B**), triglyceridemia (**C**) and triglyceridemia peak (**D**) following the ingestion of a standardized test meal challenge at lunch. Empty circles and horizontal lines within each box represent respectively the mean and median values. iAUC, incremental area under the curve; CG, chitin glucan; CTL, control. A mixed linear model for repeated measures with treatment, time, period and sequence as fixed variables and subjects as random effect has been performed. *p* values associated to CG effect assessed with the estimated treatment difference (ETD) and its 95% confidence interval are shown and considered as significant when < 0.05.

Anthropometry, body composition and fasting metabolic parameters (glycemia, insulinemia, homeostasic model assessment (HOMA), non-esterified fatty acid (NEFA), triglycerides, total, HDL and LDL cholesterol, and also resting metabolic rate were not differently impacted by CG and maltodextrin (as control) supplementations (Table S1). Compared to control, the 3-week supplementation of CG resulted in increase of the incremental area under the curve (iAUC) of postprandial exhaled H_2_ (+ 722 ppm.min) following the ingestion of a fiber-enriched breakfast. Still, compared to control, we also showed that CG decreased the iAUC of postprandial glycemia (− 60 mM.min) and triglyceridemia (− 35 mM.min) and also the peak of triglyceridemia (-0.55 mM) in response to a standardized test meal challenge served at lunch (*p* < 0.05) (Fig. 3).

### Impact of dietary interventions on gut microbiota and related metabolites

#### CG altered gut microbiota composition

After a 3-week supplementation, CG and control differently modified the relative abundance of 24 bacteria at different taxonomy levels. In particular, CG significantly decreased a family of bacteria belonging to Actinobacteria, increased *Erysipelotrichaceae* UCG.003*, Ruminococcaceae* UCG.005 and *Eubacterium ventriosum group*; while control did not impact these microbial taxa except for *Eubacterium ventriosum group* which was significantly increased (*p* < 0.05). 12 Amplicon Sequence Variants (ASVs) were differently modified by both treatments. The relative abundance of an ASV belonging to *Ruminococcus* sp. was significantly decreased after control (*p* < 0.05) whereas it remained stable after CG supplementation (Table 3). Overall gut microbiota diversity (observed OTUs, Shannon and Pielou index, Bray–Curtis distance) and biomarkers of gut barrier function (fecal zonulin and calprotectin, plasmatic LBP) were not altered (Figure S1).

**Table 3:** Effects of chitin glucan compared to control on gut microbiota composition (n = 13).

TAXA or ASV	CG			CTL			*p* value
	Before	After	*p* value	Before	After	*p* value	
			< 0.05			< 0.05	< 0.05
**Phylum**							
Proteobacteria	1.414 ± 0.707	1.255 ± 0.798		1.724 ± 1.313	1.983 ± 1.449		0.045
**Family**							
Bacteroidaceae	5.704 ± 2.656	4.941 ± 3.189		6.161 ± 3.478	6.238 ± 3.170		0.020
Unclassified Actinobacteria	**0.203** ± **0.459**	**0.016** ± **0.034**	**0.021**	**0.14** ± **0.412**	**0.093** ± **0.193**		**0.024**
**Genus**							
*Bacteroides*	5.704 ± 2.656	4.941 ± 3.189		6.161 ± 3.478	6.238 ± 3.170		0.021
*Erysipelotrichaceae.*UCG.003	**0.875** ± **0.877**	**1.303** ± **0.915**	**0.048**	**1.156** ± **0.903**	**0.745** ± **0.595**		**0.012**
*Ruminococcaceae.*UCG.005	**0.577** ± **0.516**	**1.060** ± **0.871**	**0.013**	**0.545** ± **0.533**	**0.622** ± **0.611**		**0.043**
*Lachnoclostridium*	0.661 ± 0.636	0.416 ± 0.381		0.380 ± 0.354	0.558 ± 0.403		0.007
*Barnesiella*	0.396 ± 0.326	0.354 ± 0.352		0.290 ± 0.217	0.433 ± 0.522		0.045
*Escherichia Shigella*	0.194 ± 0.280	0.182 ± 0.496		0.315 ± 0.779	0.415 ± 0.555		0.038
Unclassified Actinobacteria	0.203 ± 0.459	0.016 ± 0.034		0.140 ± 0.412	0.093 ± 0.193		0.036
*Eubacterium coprostanoligenes group*	2.029 ± 1.462	2.119 ± 1.294		2.395 ± 1.170	2.092 ± 1.229		0.038
*Eubacterium ventriosum group*	**0.349** ± **0.351**	**1.564** ± **1.232**	**0.008**	**0.285** ± **0.252**	**0.549** ± **0.496**	**0.021**	**0.014**
**ASV**							
*ASV015_Subdoligranulum sp.*	0.669 ± 0.529	0.504 ± 0.617		0.490 ± 0.605	0.539 ± 0.395		0.008
*ASV025_Faecalibacterium sp.*	0.296 ± 0.405	0.701 ± 0.564		0.580 ± 0.468	0.286 ± 0.417		0.030
*ASV027_Collinsella aerofaciens (99%)*	0.515 ± 0.385	0.444 ± 0.356		0.400 ± 0.314	0.489 ± 0.457		0.043
*ASV033_Eisenbergiella sp.*	0.594 ± 0.516	0.480 ± 0.566		0.235 ± 0.400	0.403 ± 0.508		0.045
*ASV075_Faecalibacillus intestinalis sp.*	0.121 ± 0.295	0.289 ± 0.350		0.424 ± 0.434	0.221 ± 0.372		0.007
*ASV079_Eubacterium_g23 sp.*	0.198 ± 0.324	0.297 ± 0.403		0.333 ± 0.410	0.21 ± 0.339		0.036
*ASV083_Bacteroides vulgatus (100%)*	0.235 ± 0.331	0.181 ± 0.273		0.223 ± 0.355	0.37 ± 0.447		0.028
*ASV109_Subdoligranulum variabile (99%)*	0.124 ± 0.224	0.351 ± 0.333		0.152 ± 0.217	0.181 ± 0.262		0.038
*ASV112_PAC001207_g sp.*	0.127 ± 0.276	0.236 ± 0.265		0.315 ± 0.385	0.109 ± 0.211		0.038
*ASV161_Blautia sp.*	0.127 ± 0.276	0.236 ± 0.265		0.315 ± 0.385	0.109 ± 0.211		0.021
*ASV173_Bifidobacterium adolescentis (99%)*	0.093 ± 0.157	0.096 ± 0.170		0.118 ± 0.171	0.227 ± 0.242		0.025
*ASV175_Ruminococcus sp.*	**0.000** ± **0.000**	**0.163** ± **0.319**		**0.280** ± **0.417**	**0.088** ± **0.277**	**0.025**	**0.025**

Data are expressed as mean percentage of relative abundance ± SD. To compare the shifts (i.e. the delta between the two treatments) and the baseline and endpoint, Wilcoxon signed-rank tests were performed and considered as significant if *p* < 0.05. Bacterial taxa with significant *p* inter- and intragroup are presented in bold. For ASV identification, species name are indicated when the identity is > 98%.

The concentrations of fecal metabolites (SCFA, BA, and LCFA) were not differently impacted by the two treatments (Figure S2).

### Subgroup analysis according to fermentation profile

#### The improvement in postprandial glycemic profile following the supplementation in CG is only observed in High-H_2_ group

**Figure 4:**
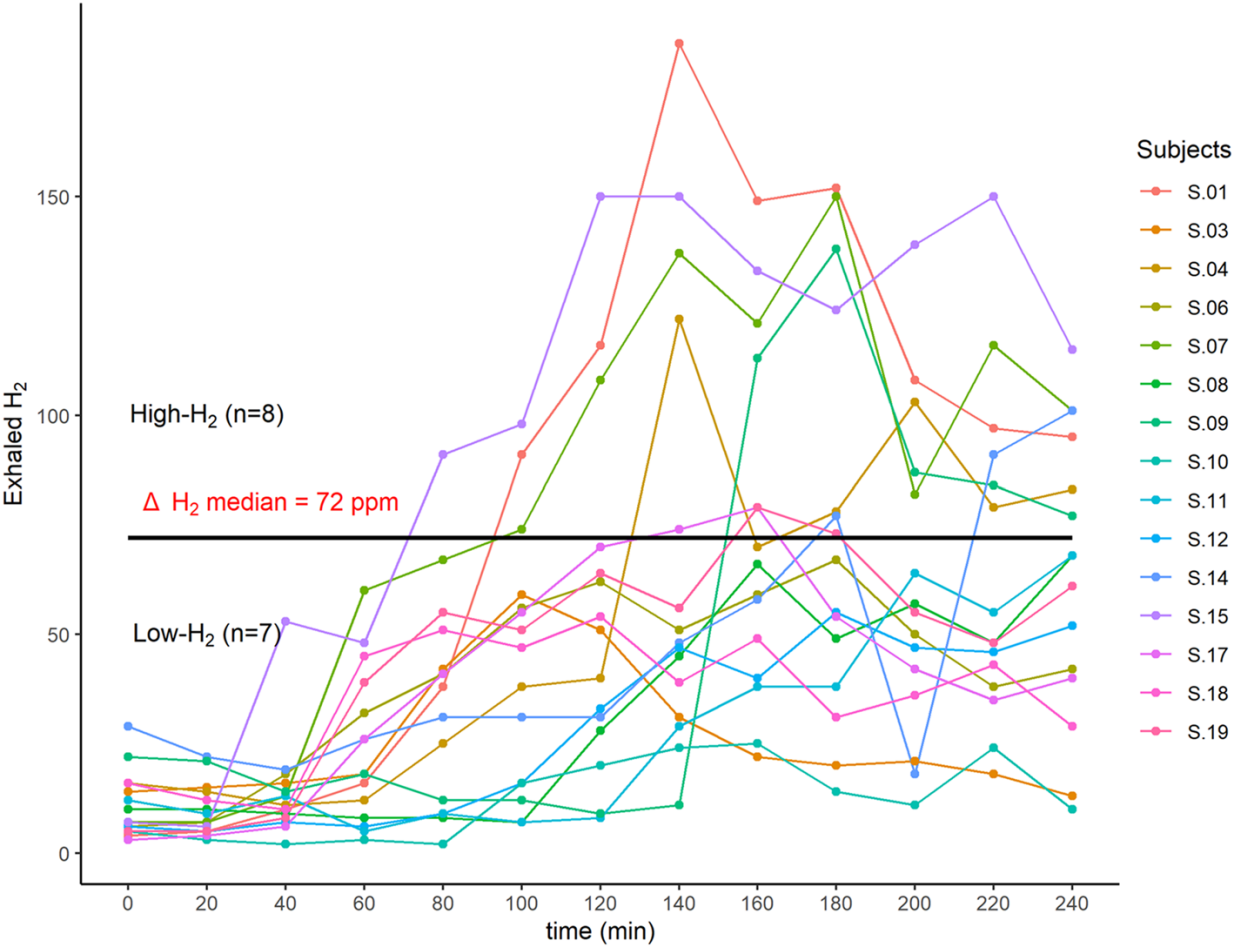
Exhaled H_2_ excretion profiles in response to lactulose breath test (n = 15). Subjects profile response to lactulose breath test from which two groups have been created: we computed Δ H_2_ = H_2_ peak − baseline; “High-H_2_” = subjects with Δ H_2_ > Δ H_2_ median; “Low-H_2_” = subjects with Δ H_2_ < Δ H_2_ median.

**Figure 5:**
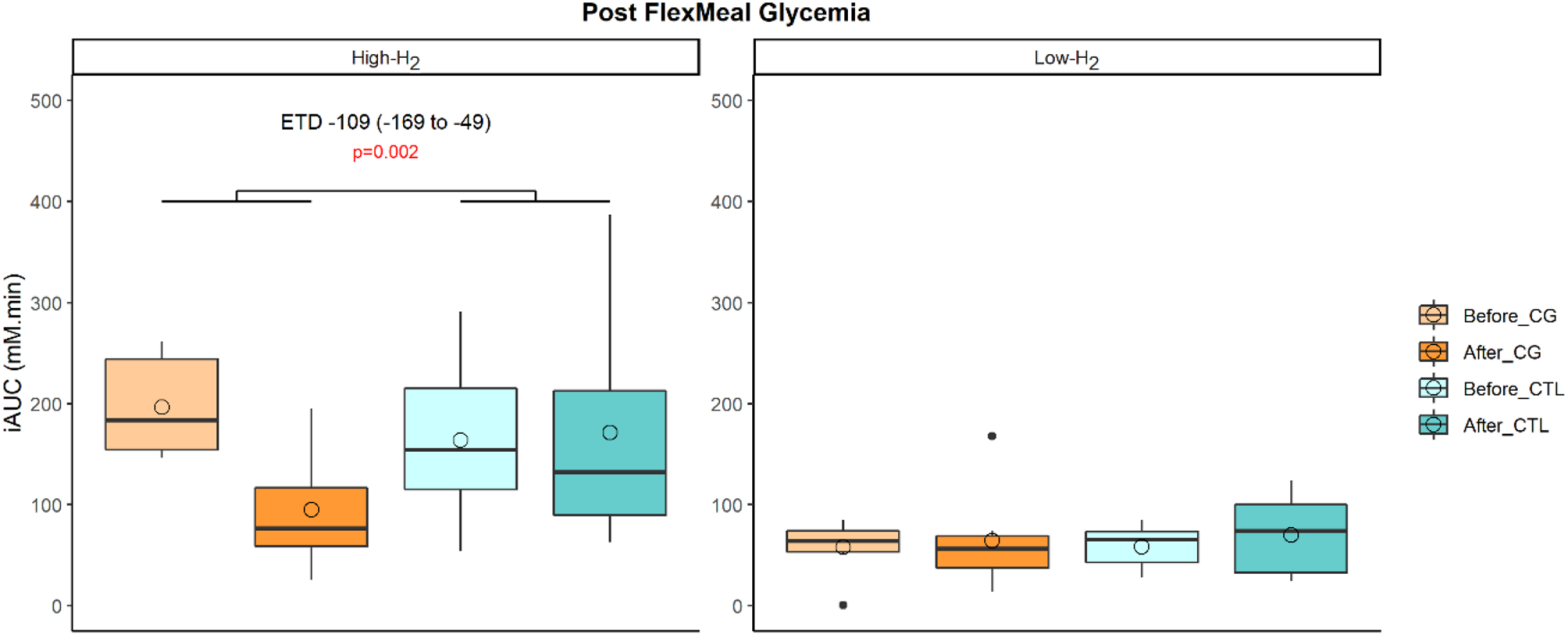
Impacted metabolic parameters according to gut fermentation profile (High-H_2_ (n = 8); Low-H_2_ (n = 7)). Empty circles and horizontal lines within each box represent respectively the mean and median values. A mixed linear model with treatment, time, period, sequence and subgroup as fixed variables and subjects as random effect has been performed once the interaction time*treatment*subgroup has been tested and showed significance. *p* values associated to CG effect assessed with the estimated treatment difference (ETD) and its 95% confidence interval is shown and considered as significant when < 0.05.

At inclusion, the results of the lactulose hydrogen breath test allowed to distinguish different profiles among the subjects: “High-H_2_” and “Low-H_2_” (Fig. 4). We then assessed if the impacts induced by the dietary interventions were different between the two subgroups. Among all assessed metabolic parameters, we found that the decrease in the iAUC of postprandial glycemia in response to the standardized test meal challenge, was only observed in “High-H_2_” subgroup (Fig. 5).

At baseline, “High-H_2_” and “Low-H_2_” subgroups differed by the fasting concentration of NEFA, the glycemic peak after the fiber-enriched breakfast, tAUC, iAUC, and peak of glycemia after the standardized test meal challenge, which were all higher in the “High-H_2_” subgroup (Table S2). Thus, subjects belonging to the “High-H_2_” group especially appeared to be presenting an impaired postprandial metabolic profile.

“High-H_2_” and “Low-H_2_” also differed at baseline in terms of abundance of some bacteria at different taxonomic levels (family, genus, ASV). Actually, compared to the “Low-H_2_” subgroup, the “High-H_2_” subgroup had a higher abundance of Actinomycetaceae, *Tyzzerela,* and a species belonging to the *PAC001207_g* genus, as well as a lower abundance of Acidaminococcaceae, Methanobacteriaceae, *Methanobrevibacter, Paraprevotella,* and *Methanobrevibacter smithii* (Table 4). Of note, bacteria with lower abundances in the “High-H_2_” subgroup were mainly methanogenic bacteria. Subgroup analysis of gut microbiota composition after the dietary interventions showed that the bacterial features differently impacted by the two treatments were not the same according to subgroups but there was no specific effect of CG supplementation (Table 5).

**Table 4:** Subgroup analysis: gut microbiota composition at baseline (n = 13).

TAXA or ASV	High-H_2_	Low-H_2_	*p* value
			< 0.05
**Family**			
Acidaminococcaceae	0.778 ± 0.793	2.032 ± 1.059	0.030
Actinomycetaceae	0.390 ± 0.247	0.129 ± 0.099	0.041
Methanobacteriaceae	0.000 ± 0.000	0.376 ± 0.430	0.021
**Genus**			
*Methanobrevibacter*	0.000 ± 0.000	0.360 ± 0.424	0.015
*Tyzzerella*	0.304 ± 0.282	0.028 ± 0.073	0.015
*Paraprevotella*	0.032 ± 0.079	0.302 ± 0.558	0.041
**ASV**			
*ASV112_PAC001207_g sp*	0.559 ± 0.389	0.071 ± 0.187	0.015
*ASV209_Methanobrevibacter smithii (99%)*	0.000 ± 0.000	0.184 ± 0.228	0.030

Gut microbiota composition differences at baseline between the two subgroups according to lactulose hydrogen breath test responses.

Data are expressed as mean percentage of relative abundance and presented as mean ± SD. Wilcoxon–Mann Whitney tests were performed and considered as significant if *p* < 0.05. For ASV identification, species name is indicated when the identity is > 98%.

**Table 5:** Subgroup analysis: Impacted microbial features (n = 13).

TAXA or ASV	CG			CTL			*p* value
	Before	After	*p* value	Before	After	*p* value	
			< 0.05			< 0.05	< 0.05
(A)							
**Family**							
Bacteroidaceae	5.995 ± 3.285	4.956 ± 3.919		5.229 ± 3.669	5.364 ± 2.743		0.022
**Genus**							
*Bacteroides*	5.995 ± 3.285	4.956 ± 3.919		5.229 ± 3.669	5.364 ± 2.743		0.036
*Erysipelotrichaceae.UCG.003*	1.150 ± 1.140	1.751 ± 0.828		1.365 ± 0.772	0.895 ± 0.764		0.035
*Lachnoclostridium*	0.967 ± 0.729	0.470 ± 0.286		0.269 ± 0.241	0.477 ± 0.394		0.022
*Butyricimonas*	0.36 ± 0.411	0.237 ± 0.468		0.175 ± 0.150	0.384 ± 0.437		0.022
*Faecalitalea*	0.376 ± 0.622	0.135 ± 0.285		0.189 ± 0.434	0.487 ± 0.911		0.022
**ASV**							
*ASV033_Eisenbergiella sp.*	0.702 ± 0.371	0.758 ± 0.564		0.245 ± 0.402	0.631 ± 0.554		0.035
*ASV045_Dorea sp.*	0.318 ± 0.499	0.832 ± 0.859		0.497 ± 0.717	0.213 ± 0.522		0.022
*ASV075_Faecalibacillus intestinalis sp.*	0.137 ± 0.335	0.279 ± 0.356		0.466 ± 0.440	0.219 ± 0.353		0.035
*ASV078_Roseburia intestinalis (99%)*	0.273 ± 0.429	0.082 ± 0.202		0.040 ± 0.098	0.499 ± 0.576		0.022
*ASV161_Blautia sp*	0.163 ± 0.399	0.157 ± 0.384		0.000 ± 0.000	0.302 ± 0.392		0.036
(B)							
**Family**							
Enterobacteriaceae	0.374 ± 0.528	0.477 ± 1.179		0.722 ± 1.868	1.115 ± 1.390		0.022
**Genus**							
*Ruminococcus*	1.165 ± 0.769	1.153 ± 0.612		1.480 ± 0.761	0.922 ± 0.943		0.035
*Coprococcus*	0.794 ± 0.535	0.636 ± 0.446		0.727 ± 0.694	0.846 ± 0.686		0.022
*Erysipelotrichaceae*	0.564 ± 1.103	0.500 ± 0.940		0.412 ± 0.705	0.639 ± 1.166		0.035
*Escherichia_Shigella*	0.274 ± 0.363	0.288 ± 0.678		0.405 ± 1.056	0.696 ± 0.623		0.022
*Slackia*	0.252 ± 0.331	0.197 ± 0.256		0.195 ± 0.280	0.276 ± 0.383		0.035
**ASV**							
*ASV015_Subdoligranulum sp.*	0.565 ± 0.620	0.343 ± 0.493		0.417 ± 0.665	0.446 ± 0.403		0.022
*ASV027_Collinsella aerofaciens (99%)*	0.510 ± 0.450	0.37 ± 0.368		0.265 ± 0.379	0.402 ± 0.620		0.036
*ASV044_Collinsella aerofaciens (99%)*	0.431 ± 0.358	0.355 ± 0.272		0.148 ± 0.269	0.262 ± 0.319	0.035	0.036
*ASV055_ [Ruminococcus] faecis (99%)*	0.546 ± 0.512	0.395 ± 0.441		0.324 ± 0.483	0.308 ± 0.385		0.022
*ASV092_ [Ruminococcus] faecis (99%)*	0.155 ± 0.410	0.129 ± 0.221		0.436 ± 0.467	0.205 ± 0.371		0.035
*ASV195_Oribacterium sp.*	0.000 ± 0.000	0.098 ± 0.260		0.055 ± 0.146	0.229 ± 0.332		0.042

(A): Impacted microbial features in “High-H_2_” subgroup.

(B): Impacted microbial features in “Low-H_2_” subgroup.

Data are expressed as mean percentage of relative abundance and presented as mean ± SD. To compare the shifts (i.e. the delta between the two treatments) and the baseline and endpoint, Wilcoxon signed-rank tests were performed and considered as significant if *p* < 0.05. For ASV identification, species name is indicated when the identity is > 98%.

### Correlation analysis between bacterial features and metabolic parameters

**Figure 6:**
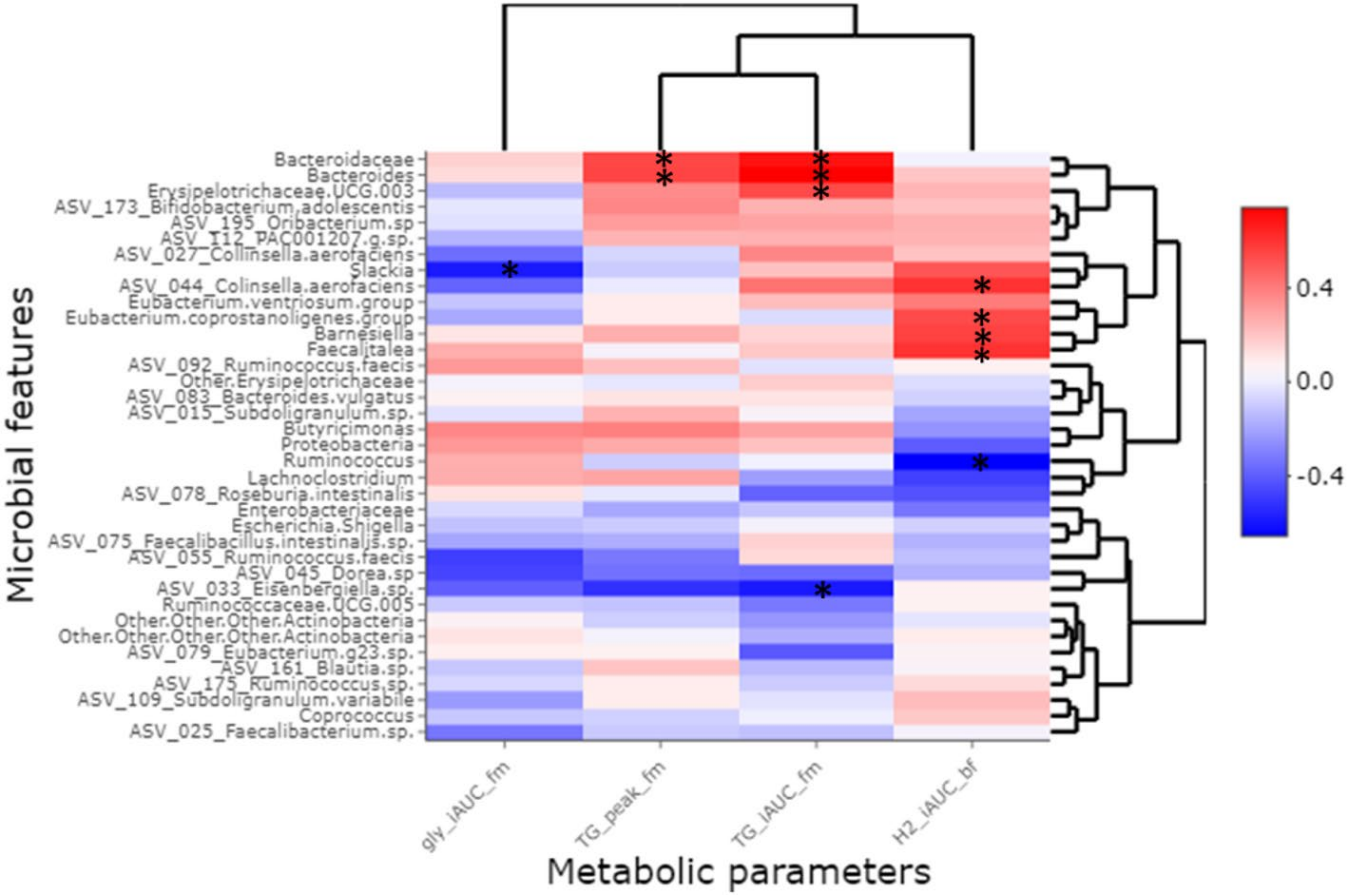
Correlations analyses between altered microbial features and metabolic parameters. The deltas (after–before) following CG supplementation for each metabolic parameter and microbial feature which were differently impacted by the two interventions (including those impacted in subgroup analysis) are presented. iAUC: incremental area under the curve; gly: glycemia; TG: triglyceridemia; H2: hydrogen; bf: standardized breakfast; fm: Flexmeal (standardized test meal challenge). Spearman’s correlations were performed. * indicate the significant correlations (*p* value < 0.05).

To assess the relationship between gut microbiota and cardiometabolic profile alterations, we performed a correlation analysis between bacterial features and metabolic parameters which were differently altered by the dietary interventions (Fig. 6). Correlation analyses showed that the iAUC of exhaled H_2_ after the fiber-enriched breakfast correlated with *Eubacterium coprostaligenes, Barnesiella, Faecalitalea* and ASV belonging to *Colinsella aerofaciens*, and inversely correlated with *Ruminococcus*. The iAUC of glycemia after the standardized test meal challenge inversely correlated with *Slackia*. The iAUC of triglyceridemia after the standardized test meal challenge positively correlated with Bacteroidaceae, *Bacteroides* and *Erysipelotrichaceae* UCG.003*,* and inversely correlated with an ASV belonging to *Eisenbergiella* sp. *Bacteroides* and Bacteroidaceae also positively correlated with the peak of triglyceridemia after the standardized test meal challenge.

## Discussion

In this exploratory study performed in a population of subjects at CMR of whom 90% were dyslipidemic, we showed that compared to maltodextrin as control, 3-week supplementation with CG modified the gut microbiota composition by changing the relative abundance of several gut bacterial taxa. This effect was accompanied by a significant improvement of postprandial glucose and lipid profiles.

The effects of CG supplementation on gut microbiota and bacterial co-metabolites in humans has been recently studied in healthy subjects, revealing that CG increased the relative abundance of the butyrate-producing bacteria *Roseburia* sp, and coherently increase SCFA production including butyric acid^15^. In our study, *Roseburia* sp abundance and fecal SCFA concentrations were not differently modified by the two treatments, even if butyric acid tended to be higher after CG supplementation. These results suggest that the production of metabolites could differ according to the metabolic status of participants or to their gut microbiota characteristics. It is also well known that the pathways of metabolites’ production are diverse and redundant among the different microbiota species. It is therefore possible that the impacted microbial features may be involved in opposite pathways with the resultant corresponding to the “no effect” we observed when measuring the metabolites.

In this study, we also showed that exhaled H_2_ following the intake of a fiber-enriched breakfast was increased by CG compared to control, suggesting the ability of CG to induce a higher fermentation in response to the dietary fiber intake.

Interestingly, still compared to control, CG improved postprandial glucose and lipid metabolism as illustrated by the decrease in iAUC of glycemia and triglyceridemia, but also the peak of triglyceridemia in response to a standardized test meal challenge, the FlexMeal. In a 6-week, randomized, double-blind and placebo-controlled study conducted in healthy overweighed volunteers, the same dose of CG also exhibited beneficial effects by reducing blood levels of oxidized low-density lipoproteins (ox-LDL)^16^. We did not observe such effect here, possibly due to the difference in terms of study duration. In accordance with the effects observed on postprandial glycemic and lipemic profiles in this study, it has been previously shown that in mice fed with a high-fat diet (HFD), a 4-week administration of CG significantly improved metabolic parameters, including glucose tolerance^12^. Another in vivo study also demonstrated beneficial effects of CG in lowering plasma triglycerides in hamsters fed with an atherogenic diet but at fasting state^11^. Presently, the observation of effects at postprandial and not at fasting state, bolsters the interest of postprandial phase when assessing the effects of nutritional interventions and more generally human health^17^. Furthermore, postprandial hyperglycemia and hyperlipemia are now recognized as independent cardiometabolic risk factors^18^. An exacerbated and prolonged postprandial lipemia is typically observed in subjects with metabolic disorders^19^. Also, epidemiological data reported that post-challenge glycemia is associated with cardiovascular risk, regardless of fasting biomarkers such as fasting glycemia and glycated hemoglobin (HbA1c) and even has a greater predictive power of cardiovascular events^20^. Indeed, abnormally increased levels of glucose and lipids generates excessive amounts of free radicals (or reactive oxygen species triggering a biochemical cascade throughout the circulation, inducing inflammation, endothelial dysfunction, hypercoagulability, sympathetic hyperactivity and a more atherogenic lipoprotein profile^21–23^). Therefore, a reduction of postprandial hyperglycemia and hyperlipemia excursions, as demonstrated in the present study with 3-week supplementation of CG, is of interest to improve cardiometabolic profiles.

H_2_ has been reported to have antioxidant, anti-inflammatory, and anti-apoptotic properties^24^. In patients with type 2 diabetes or impaired glucose tolerance, supplementation of H_2_-rich water improved lipid and glucose metabolism^25^. Similarly, in db/db mice, drinking H_2_-water has been shown to decrease levels of plasma glucose, insulin, and triglyceride, the effect of which on hyperglycemia was similar to diet restriction^26^. The suggested underlying mechanism is an observed increase of the expression of a hepatic hormone, fibroblast growth factor 21 (FGF21), which is a regulator of energy expenditure. Here, the energy metabolism as measured by resting metabolic rate did not show significant differences between the two treatments. Subgroup analyses revealed that the improvement in postprandial glucose metabolism, especially the decrease in the iAUC of postprandial blood glucose, was only observed in the “High-H_2_” subgroup composed of subjects with a higher concentration of exhaled H_2_ in response to the lactulose H_2_ breath test. This study is the first of its kind to identify the production of H_2_ as an indicator of a fiber-derived impact on glucose metabolism. “High H_2_” subjects had a lower abundance of methanogenic bacteria including *Methanobrevibacter smithii*, which is the most dominant methanogen in the gastrointestinal tract in humans^27^. It has been reported that methanogens increase in ob/ob mice^28^ and that in obese humans CH_4_ production is associated with a higher BMI^29^. The suggested mechanism is that methanogens use the H_2_ produced by syntrophic organisms for their own anaerobic metabolism, after which CH_4_ is produced as a by-product. This H_2_ scavenging allows syntrophic organisms to be more productive, increasing SCFA production and availability of calories for the host^30,31^. Subjects included in our study were all H_2_ producers and even if the comparison between the two subgroups showed that they differed by their abundance in methanogenic bacteria, no significant difference in terms of exhaled CH_4_ was found. Interestingly, at baseline, when firstly challenged with the standardized test meal, subjects belonging to the “High-H_2_^”^ subgroup were the ones who presented a higher fasting concentration of NEFA and glycemia peak after the fiber-enriched breakfast, tAUC, iAUC and peak of glycemia after the standardized test meal challenge. Thus, the subjects who have benefited the most from the intervention in terms of glycemic profile improvement would actually be those who already have impaired postprandial metabolic profiles.

Of note, betaglucan (BG) is one component of CG. It has been shown to decrease postprandial glycemia after an oral glucose test tolerance (OGTT) or when added to food products (pasta, bread, soup, cereals, etc.) in healthy or diabetic subjects^32–36^. Their postprandial glucose lowering effect has long been reported to be essentially due to a delayed and somewhat reduced carbohydrate absorption from the gut^37,38^. However, since different studies have shown BG impacts on gut microbiota without assessing in parallel the impacts on glycemic response, other mechanisms may exist but need further investigations to be elucidated^39^.

When assessing the link between impacted metabolic and bacterial parameters, Slackia was the only bacterial feature which showed direct correlation with postprandial glycemia. It has been reported that the abundance of this genus was higher in prediabetic subjects and reduced after supplementation in xylooligosaccharide^40^. Here, we showed that this genus was differently impacted by the two treatments and particularly decreased by CG even if not statistically significant and only in « Low-H_2_». The improvement of postprandial triglycerides metabolism with CG supplementation was correlated with the decrease in rather pro inflammatory microbial features. The correlation analyses also suggested that the increase of exhaled H_2_ after the fiber-enriched breakfast may be explained by abundance’s modification of a cluster of microbial features including butyrate-producers. Consistently, a link between bacterial substrate metabolism and H_2_ gas formation have already been proposed in butyrate-producing colon bacteria^41,42^.

Our study presents some strengths and limitations. We assessed a cross-over design study with a comprehensive set of biomarkers from gut microbiota composition and functions to fasting and postprandial phenotyping. Even though 4.5 g chitin-glucan were not sufficient to increase SCFA or barrier function biomarkers, it allowed to improve postprandial metabolism, which has been reported to be associated to cardiometabolic risk profile. For the first time, we identified an interesting non-invasive biomarker: exhaled H_2_ following a lactulose breath test, which could be of interest to evaluate dietary fiber impact on postprandial glucose metabolism. We also showed that CG supplementation increased exhaled H_2_ following a fiber-enriched breakfast. Although several beneficial metabolic effects of H_2_ enrichment have been reported (antioxidant, anti-inflammatory, and anti-apoptotic properties, lipid and glucose metabolism improvement)^24–26^, we cannot say if the present H_2_ could have the same effects. The limitations of this study is that it was a short-term exploratory study performed in a small number of participants, with a comprehensive set of biomarkers so that after adjustment for multiple comparisons, none of the *p* values remain significant. Due to the explorative character of the study, we thus presented and considered non-adjusted *p* values < 0.05 as significant.

To conclude, this study showed the potential effects of a 3-week supplementation of CG compared to maltodextrin in at cardiometabolic subjects. No specific fecal biomarkers of CG interactions with the gut microbiota (as shown in the study in healthy volunteers) has been demonstrated in the present study. However, presently the improvement in the postprandial glycemic and lipemic profiles, early determining markers of cardiometabolic risk, underlies the interest of such dietary intervention in this specific at-risk population. The profile response to lactulose H_2_ breath test seems to be a parameter of interest to predict the effectiveness of the intervention especially in terms of glycemic response. Further investigations are needed to confirm these observations and to elucidate the underlying mechanisms of CG on cardiometabolic profile in subjects at cardiometabolic risk.

## Materials and methods

### Ethical statements

This study was reviewed and approved by the local ethical committee CPP Ile de France IV 2018/69, ID-RCB: 2018-A02155-50. It was conducted between November 2018 and June 2019 at the Human Nutrition Research Center of Rhône-Alpes (CRNH-RA) and carried out in accordance with the Second Declaration of Helsinki and French Jardé’s law. It was reported and registered on http://www.clinicaltrials.gov NCT03773900 (12/12/2018). All participants received and signed informed consent before the initiation of any study-related procedure.

### Study participants

Sixteen subjects with CM risk profile were recruited. Inclusion criteria included age: 30–65 years old, BMI: 25–35 kg/m^2^, waist circumference > 80 cm for women and > 94 cm for men, daily fiber consumption < 25 g/day, exhaled H_2_ (max–min > 20 ppm) in response to lactulose test, stable weight and moderate physical activity, no known gastrointestinal disease, no previously bariatric surgery, no use of antibiotics or other drugs interfering with microbiota composition in the 3 months prior to the beginning of the study.

### Study design

This was a dietary monocentric, randomized, double blind and cross-over study. Each subject received two 3-week dietary interventions in a random order according to a randomization list per block of permutations established with SAS software 9.4 by the statisticians. The two dietary interventions were, 1) 4.5 g/d of chitin-glucan (CG) and 2) 4.5 g of maltodextrin as control (Table S3). A 4 to 6-week washout period separated the two interventions. Before and after each intervention, fecal samples were collected for gut microbiota composition and its derived-metabolites ‘analysis. Fasting and postprandial metabolic parameters were evaluated during metabolic assessment days (Figure S3).

CG and CTL had the same galenic form and visual aspect but mainly differed by their fiber content (Table S3). Both were presented as a 3 g sachet powder to be diluted in water. Each sachet included 1.5 g of CG or 1.5 g control, thus subjects consumed 3 sachets per day (20–30 min before the 3 meals of the day) to reach the target dose of 4.5 g/d. The set of sachets to be consumed for each period was given to subjects at the beginning of each period during which, they were asked to keep the same diet and usual physical activity. A French National Authority for Health (HAS) questionnaire assessed with NUTRILOG software (version 3.10b, released in February 2017) was used to evaluate subjects’ dietary fiber intake at inclusion. To evaluate dietary intake and ensure a good compliance during the intervention periods, subjects were ordered to fill in a 3-day dietary record. Data from this 3-day dietary record were also processed using NUTRILOG software which allowed to estimate energy, macronutrient, and dietary fiber intakes. Study’s products consumption compliance was evaluated by counting full and empty sachets they brought to the center at the end of every intervention period.

During the 3 days before metabolic assessment day, subjects were ordered to collect stool samples using a specific kit. Stool samples were kept in the subject’s freezer until they brought them to the center using a furnished cooler bag. On metabolic assessment days, subjects arrived at CRNH-RA after an 8-h overnight fast following the ingestion of a standardized low dietary fiber evening meal (one serving of fish, rice, cheese, rusk or soft bread, dried biscuits and butter). Body weight and fat mass percentage were measured using standardized methodologies using a calibrated weighing scale and a Bodystat Quadscan 4000 (BQ4000; Bodystat Ltd. Douglas, UK) respectively. RMR was measured by indirect calorimetry using a QUARK calorimeter (Cosmed, Rome, Italy). Subjects were served a dietary fiber-enriched breakfast at T0 (rye bread, hazelnut carob spread, fruit juice) and a standardized challenge test meal: the FlexMeal challenge at T240. The FlexMeal is a revised version of the “PhenFlex drink”^43^. It consists on a standardized challenge test meal composed by a soup and a dairy dessert presenting 923 kcal, 32.5% of carbohydrates, 58.9% of lipids and 8.7% of proteins. Fasting and 4 h postprandial blood (T0, T30, T60, T90, T120, T180, T240, T255, T270, T300, T330, T360, T420) were collected using an antecubital vein catheter. Fasting and postprandial exhaled gases were also collected (T0, T60, T120, T180, T240, T300, T360, T420, T480, T540, T600).

### Biochemical blood analyses

Collected blood was centrifuged immediately for 10 min at 4500 rpm. Plasma was stored at − 20 °C until the assays were conducted.

Glycemia was measured by spectrophotometry according to Architect Abbott Hexokinase method; TC, HDL-C and TG by spectrometry using Architect Module Chimie Abbott method; insulin by radio immunoassay according to RIA CisBio IBA method, NEFA by automatized spectrometry with PENTRA 400 HORIBA ABX. LDL-C was calculated using Friedwald formula and HOMA as plasma glucose (mmol/L) x plasma insulin (mUI/L) / 22.5.

### Exhaled gases analyses

Exhaled gases have been collected using EASYSAMPLER Breath Test Kit (Quintron, Milwaukee, WI, USA). The concentrations of H_2_, CH_4_ and CO_2_ were then measured by gas chromatography using a QUINTRON BREATH TRACKER analyzer (Quintron, Milwaukee, WI, USA).

### Microbiota analysis

#### Fecal microbiome sequencing analysis

Bacterial DNA was extracted from fecal samples using the QIAamp DNA Stool Mini Kit (QIAGEN, Hilden, Germany), following the ‘Protocol Q’ that was described by Costea and colleagues^44^ with a slight modification: a reduction in time of bead beating step. Cells are mechanically lysed by running the FASTPREP Instrument for 2 min at max speed (beating 1 min and resting 5 min).

Library construction and the Illumina sequencing protocol has been previously described in detail^45^. In brief, the V5-V6 regions of the 16S rRNA gene were targeted for PCR amplification using primers 784 F [5′-RGGATTAGATACCC-3′] and 1064 R [5′- CGACRRCCATGCANCACCT-3′]. 16S rRNA gene amplicons were sequenced by the MiSeq platform (300 bp paired-end length) at the University of Minnesota Genomics Center. All samples of this study were sequenced in the same run. Paired-end reads were merged, demultiplexed and conducted quality control implementation (length = 281 bp, mean sequence quality score ≥ 30) using QIIME2 pipeline with DADA2^46,47^, which we refer to herein as amplicon sequences variants (ASV). The average of the sequence in all samples (n = 60) was 19,884. An even depth of 8,290 sequences per sample was used to conduct microbiome diversity. We assigned the sequences to taxonomic categories including kingdom, phylum, class, order, family and genus levels using a pre-trained Naive Bayes classifier based on Silva 132 99% OTUs database^48^. For significant ASV, in order to have a higher resolution of the ASV identification, online 16S rRNA databases on both NCBI blast and EzBioCloud platforms were also used.

### SCFA analysis

The methodology has been described before^15^. In brief, the native fecal samples were homogenized and subsamples of 400–500 mg were diluted 1:4 in ultrapure water and analyzed using a capillary gas chromatograph (HP6890 Series; Hewlett Packard Corp., Paolo Alto, California, USA). Fecal dry mass was assessed by drying 300–500 mg of native sample overnight at 103 °C.

### Markers of intestinal permeability

All biomarkers included were validated as markers of intestinal permeability before (https://pubmed.ncbi.nlm.nih.gov/34009040/). Zonulin and calprotectin were measured using enzyme-linked immunosorbent assay kits (K5600; K6927; Immundiagnostik AG, Bensheim, Germany) following the manufacturer’s protocol. The fecal samples were diluted to the working concentration in sample buffer using stool sample tubes (K6998SAS; Immundiagnostik AG, Bensheim, Germany). LBP (REFs: DY870-05 and DY008; Bio-Techne GmbH, Wiesbaden, Germany) was measured in plasma.

### Bile acids analysis

Bile acids were analyzed using method validated method adapted from *Guillemot-Legris *et al.^49^. Briefly, lyophilized feces (5 mg) were homogenized in ice-cold distilled water prior to protein precipitation using acetone containing seven deuterated bile acids used as internal standards. Samples were next centrifuged, and the supernatant was evaporated to dryness under nitrogen steam. The resulting residue was resuspended in methanol and injected in the HPLC–MS system consisting of an LTQ-Orbitrap XL mass spectrometer (Thermo Fisher Scientific) coupled to an Accela HPLC system (Thermo Fisher Scientific). Analyte separation was performed on an Ascentis Express C-18 column (2.7 µm, 100 × 4.6 mm) (Sigma-Aldrich) using a gradient between acetonitrile and water, both containing formic acid. Mass spectrometry analysis was performed using an electrospray ionization source in the negative mode. Calibration curves were prepared in the same conditions.

### LCFA analysis

To determine the LCFA profile in feces, we used 40 mg of previously lyophilized feces during 48 h (Labconco, freeze dryer 4.5). Forty microliter of C19 were added as extraction standard for homogenization with methanol:chloroform (1:2 V/V) by brief sonication in ice (Labsonic U, B. Braun). Homogenates were then filtered with Whatman filters n°1 (10 μm of porosity). Filters were rinsed with 2 ml of chloroform and 1 ml of methanol. Homogenates were purified with KCl 0.88% and KCl 0.88%: methanol (1:1 V/V). After centrifugation (1500 g, 5 min), the chloroform phase was collected in new tubes and evaporated under nitrogen flux until samples were completely dry. The esterified fatty acids were then subjected to alkaline hydrolysis (saponification) and free fatty acids were methylated and quantified by gas chromatography with flame ionization detector as previously described^50^.

### Statistics

The primary outcome of this study was the measure of SCFA production. Due to the lack of available published data on this specific fiber, a standard power calculation to obtain the number of subjects needed for the study was not possible. The choice of the primary outcome relayed on data from two studies. The first one, carried out in an in vitro model simulating the intestine showed that a dose of 4.5 g of CG was able to increase the production of SCFA^13^. The second one showed that a supplementation of 3 g/day for 2 months of BG, a component of CG and a dietary fiber which structure is closely related CG, resulted in a significant increase of SCFA in the feces of 26 subjects^51^.

All statistical analyses and graphs were performed with R software v3.6.0 and SAS software 9.4 TS Level 1M6^52^.

To find out whether CG had a different effect from the effect of control on clinical variables and to assess if the difference was statistically significant, we performed a linear mixed model for repeated measures, with Toeplitz or autoregressive structure (AR) as covariance structure. In order to account for variability between subjects and to adjust for any non-specific differences, subjects were included as random effects. To quantify the effect of CG compared to the effect of control, we estimated the difference in terms of change ETD = After Fiber − Before Fiber − (After Control − Before Control) to which the *p* value of the interaction treatment*time is associated.

For gut microbiota analysis, relative abundances performed in Qiime2 were calculated in R for each taxa and ASV. To avoid analyzing spurious sequences, ASV with an average relative abundance below 0.1% in all samples were removed. The same cut-off was applied for analysis of bacterial genera. To assess if a given microbial feature was differently impacted by the two treatments, we first computed the delta (after–before) in abundance for both treatments. Then, a Wilcoxon signed-rank test has been performed to assess if the difference between the two deltas was statistically significant; if so, we evaluated the impact of each treatment by comparing the abundances before and after its consumption with a Wilcoxon signed-rank test.

Beta-diversity indices were compared by PERMANOVA using the Adonis function in the vegan package on R Software and data were visualized using non-metric multidimensional scaling.

For subgroup analysis, we computed Δ exhaled H_2_ = exhaled H_2_ peak – baseline and defined two subgroups: “High-H_2_” = subjects with Δ exhaled H_2_ > Δ exhaled H_2_ median and “Low-H_2_” = subjects with Δ exhaled H_2_ < Δ exhaled H_2_ median. The interaction treatment*time*subgroup was tested for all variables and the linear mixed model for repeated measures was only performed when the interaction was significant (*p* < 0.05).

For correlation analyses, we computed the deltas (after–before) following CG supplementation for each metabolic parameter and bacterial feature which were differently impacted by the two intervention. Spearman’s correlations were performed.

All statistical analyses were performed with adjustment using false discovery rate (FDR < 0.05) for multiple tests according to the Benjamini–Hochberg procedure and none of the adjusted *p* values were significant. Nevertheless, non-adjusted *p* values < 0.05 were considered significant due to the explorative character of these approaches.

## Supplementary Information

Supplementary Information.

## Data Availability

The raw sequencing data are deposited into the Sequence Read Archive (SRA) of NCBI (http://www.ncbi.nlm.nih.gov/sra) under BioProject PRJNA803331. The study protocol and the datasets generated during and/or analyzed during the current study, including deidentified participant data will be available from the corresponding author on reasonable request.
