# Supplementary material for: Chitin-glucan supplementation improved postprandial metabolism and altered gut microbiota in subjects at cardiometabolic risk in a randomized trial
Source: Sci Rep. 2022 May 25;12:8830. doi: 10.1038/s41598-022-12920-z (PMC9132890; doi:10.1038/s41598-022-12920-z)
Supplement: Supplementary file 1 — Supplementary Information. [file 41598_2022_12920_MOESM1_ESM.docx]

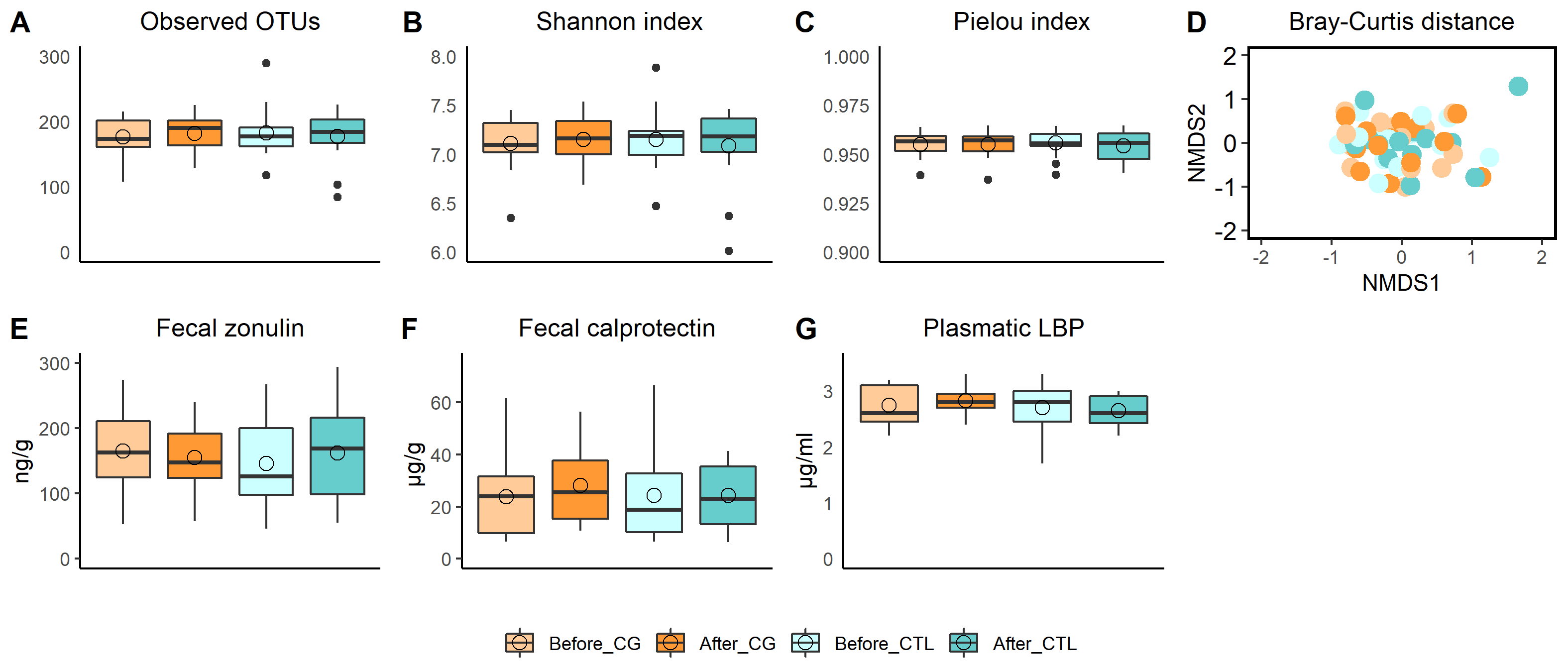


**Figure S1**. Gut microbiota diversity and biomarkers of gut barrier function

A-C: measures of α-diversity: Observed OTUs, Shannon and Pielou’s indexes (n=13)

D: Non-metric multidimensional scaling of the β-diversity index Bray-Curtis (n=13)

E-F: fecal concentrations of zonulin and calprotectin (n=15)

G: plasmatic concentration of LBP (n=15)

LBP: Lipopolysaccharide Binding Protein.

Empty circles and horizontal lines within each box represent respectively the mean and median values. None of the p-values associated to CG effect assessed with the estimated treatment difference (ETD) or Permanova test was significant.

**Figure S2**. Gut bacterial co-metabolites (n=15)

A: Fecal concentrations of SCFA.

B: Fecal concentrations of BA.

C: Fecal concentrations of LCFA.

Data are normalized to the mass of dry matter and are expressed as mean ± SD. SCFA: short-chain fatty acids; BA: bile acids; LCFA: long-chain fatty acids. A mixed linear model for repeated measures with treatment, time, period and sequence as fixed variables and subjects as random effect has been performed. None of the p-values associated to CG effect assessed with the estimated treatment difference (ETD) was significant.


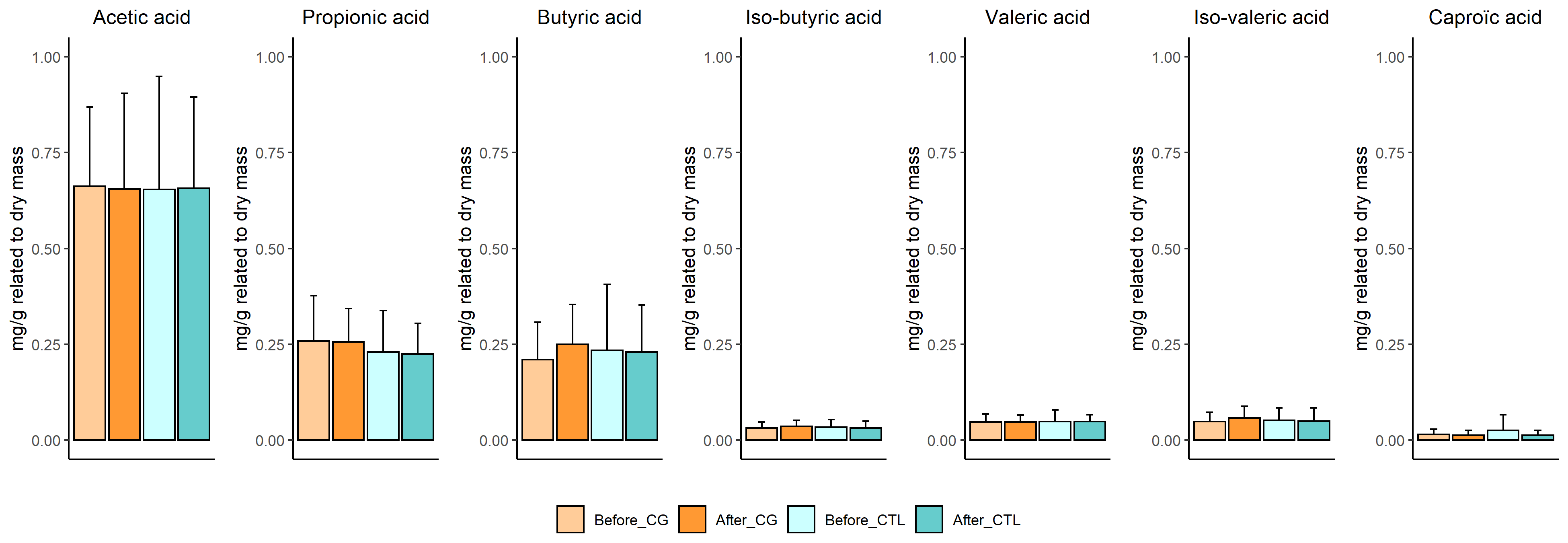


**A**


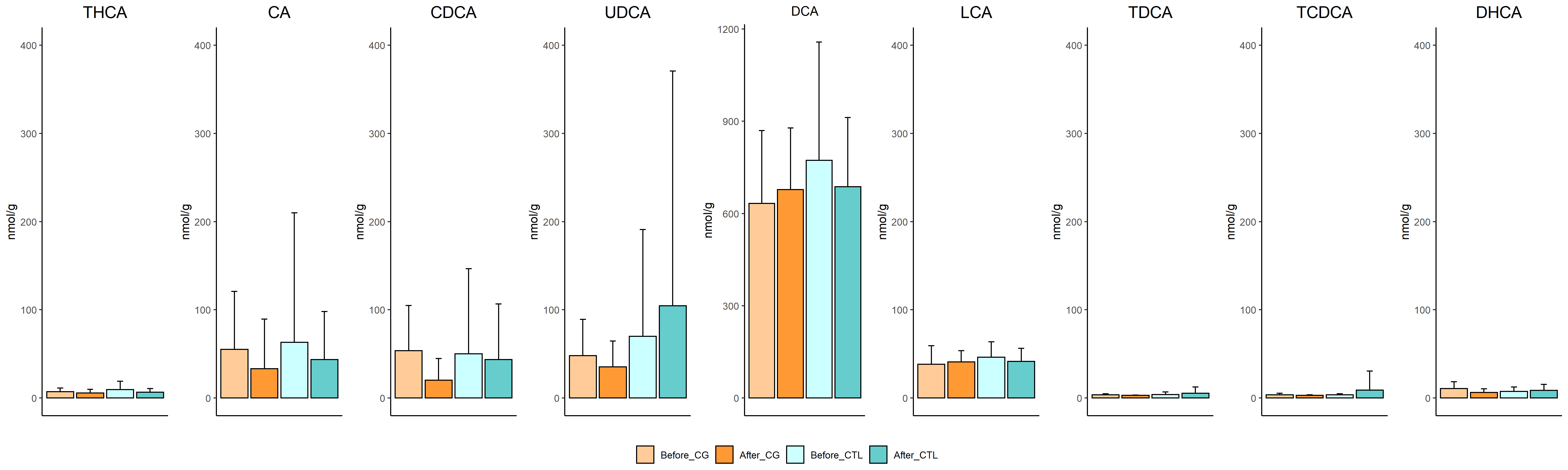


**B**

**C**


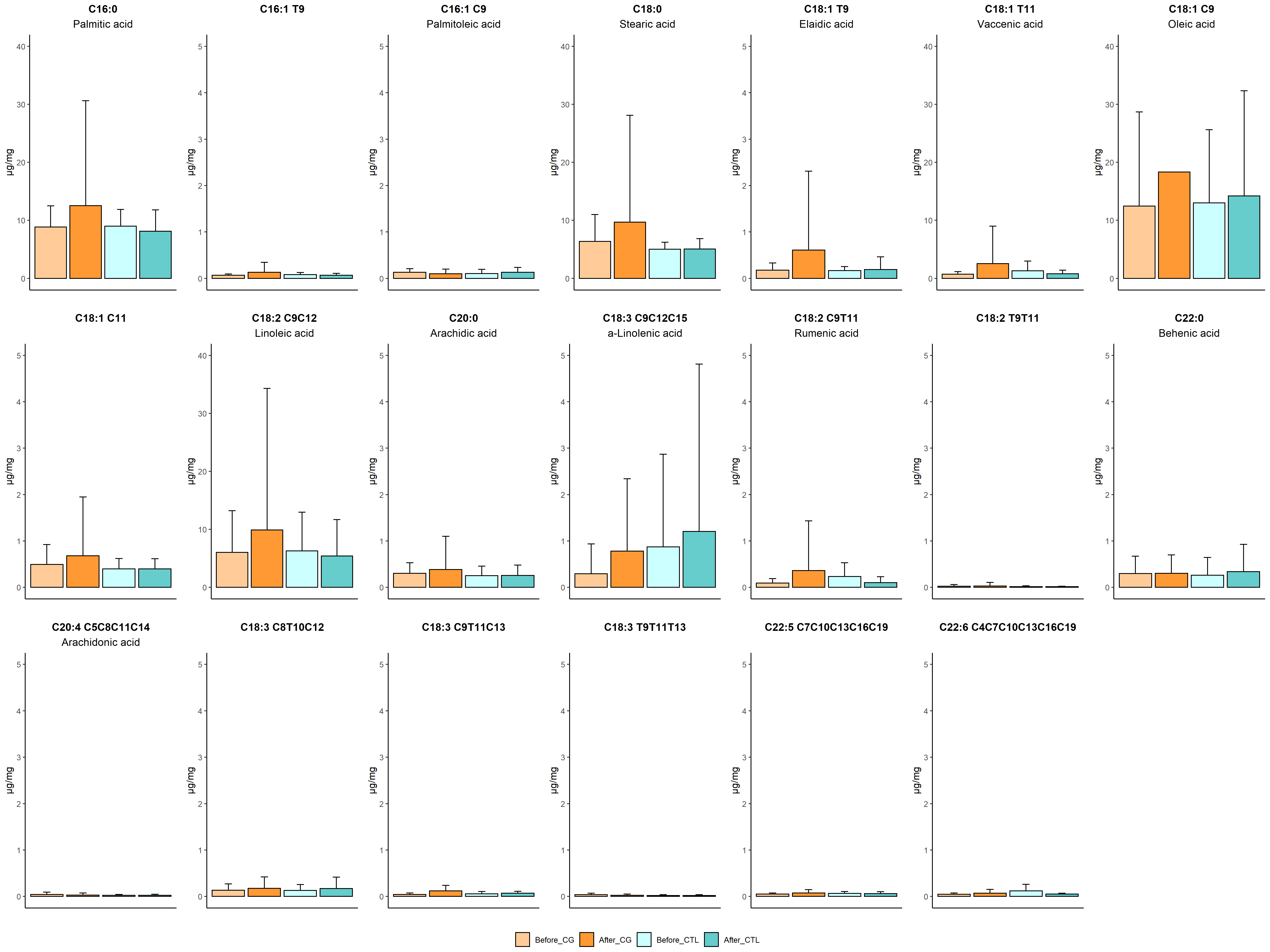


**
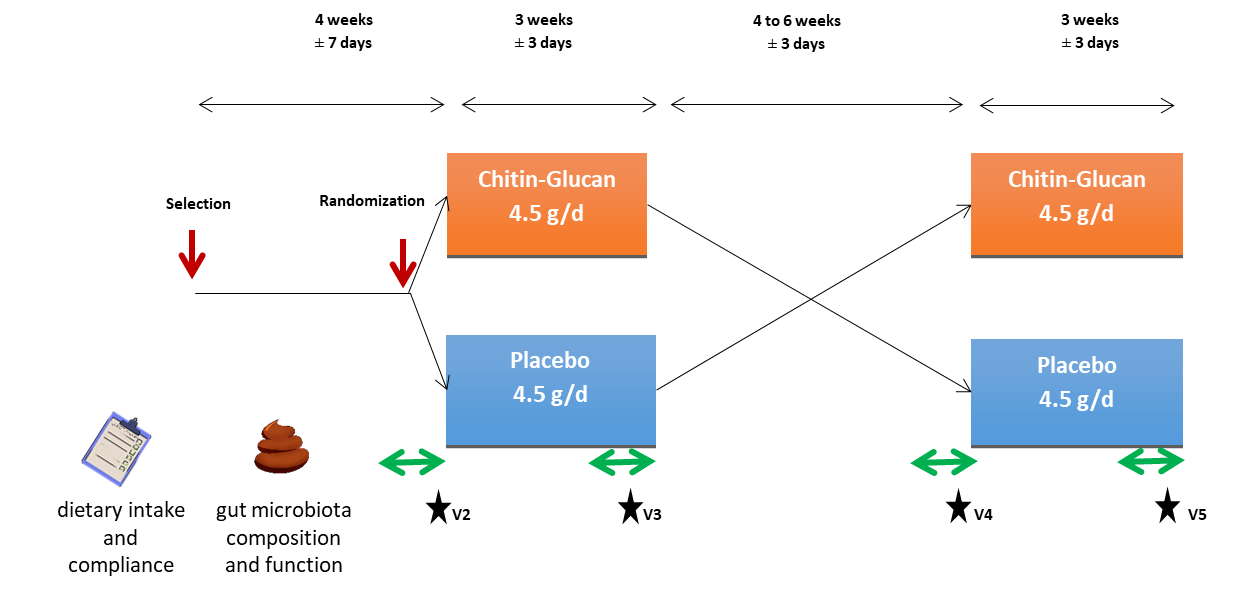
**

**
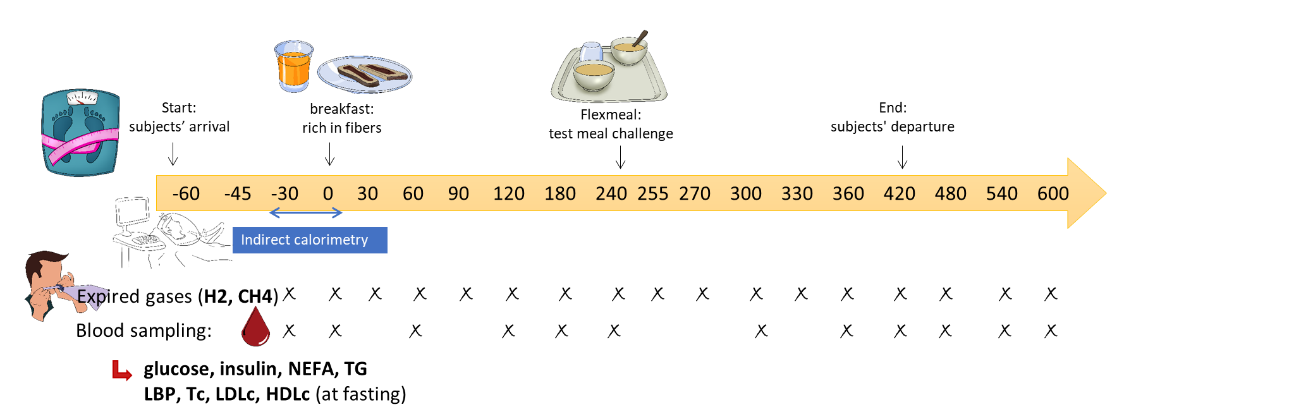
**

**Figure S3**. Protocol design of the study and metabolic assessment days.

|  | **CG** | | | | | | | **CTL** | | | | | | | | | **ETD** | | |  |
| --- | --- | --- | --- | --- | --- | --- | --- | --- | --- | --- | --- | --- | --- | --- | --- | --- | --- | --- | --- | --- |
|  | **Before** | | | **After** | | | | **Before** | | | | | **After** | | | | | **p-value** | | |
| Weight (kg) | 83.14 | ± | 12.73 | | 82.92 | ± | 12.48 | | 83.17 | ± | 12.34 | 83.17 | | ± | 12.61 | ns | | |  |  |
| BMI (kg/m^2^) | 28.55 | ± | 5.13 | | 28.45 | ± | 4.88 | | 28.58 | ± | 5.16 | 28.57 | | ± | 5.23 | ns | | |  |  |
| Fat mass (%) | 30.72 | ± | 8.15 | | 30.76 | ± | 8.42 | | 30.64 | ± | 8.23 | 30.86 | | ± | 8.12 | ns | | |  |  |
| Glycemia (mM) | 5.38 | ± | 0.39 | | 5.49 | ± | 0.44 | | 5.51 | ± | 0.32 | 5.51 | | ± | 0.37 | ns | | |  |  |
| Insulinemia (mIU/l) | 11.57 | ± | 4.59 | | 10.8 | ± | 5.48 | | 10.02 | ± | 2.07 | 11.35 | | ± | 5.49 | ns | | |  |  |
| HOMA | 2.77 | ± | 1.13 | | 2.67 | ± | 1.45 | | 2.46 | ± | 0.58 | 2.81 | | ± | 1.48 | ns | | |  |  |
| NEFA (µM) | 483 | ± | 167 | | 497 | ± | 218 | | 509 | ± | 139 | 445 | | ± | 149 | ns | | |  |  |
| TG (mM) | 1.14 | ± | 0.35 | | 1.1 | ± | 0.38 | | 0.98 | ± | 0.32 | 1.07 | | ± | 0.42 | ns | | |  |  |
| TC (mM) | 4.49 | ± | 0.76 | | 4.94 | ± | 0.76 | | 4.72 | ± | 0.66 | 4.81 | | ± | 0.72 | ns | | |  |  |
| HDL-c (mM) | 1.12 | ± | 0.22 | | 1.53 | ± | 1.05 | | 1.09 | ± | 0.25 | 1.12 | | ± | 0.2 | ns | | |  |  |
| LDL-c (mM) | 3.15 | ± | 0.73 | | 2.91 | ± | 0.93 | | 3.2 | ± | 0.61 | 3.21 | | ± | 0.65 | ns | | |  |  |
| RMR (kcal/24h) | 1629 | ± | 277 | | 1674 | ± | 357 | | 1656 | ± | 319 | 1626 | | ± | 263 | ns | | |  |  |

**Table S1**. Effects of chitin glucan compared to control on anthropometry, body composition and fasting metabolic parameters (n=15)

Data are expressed as mean ± SD. CG: chitin glucan; CTL: control; BMI: body mass index; HOMA: homeostasic model assessment; NEFA: non-esterified fatty acid; TG: triglycerides; TC: total cholesterol; HDL cholesterol: high-density lipoprotein cholesterol; LDL-C: low-density lipoprotein cholesterol; RMR: resting metabolic rate. A mixed linear model for repeated measures with treatment, time, period and sequence as fixed variables and subjects as random effect has been performed. Unadjusted p-values associated to CG effect assessed with the estimated treatment difference (ETD) are shown and considered as significant when <0.05.

|  | **High-H_2_ (n=8)** | | | **Low-H_2_ (n=7)** | | | | **p value <0.05** |
| --- | --- | --- | --- | --- | --- | --- | --- | --- |
| exhaled H_2_ (lactulose breath test) (ppm) | 119 | ± | 33.34 | | 50.88 | ± | 16.74 | 0.01 |
| α linolenic acid (µg/ mg) | 0.08 | ± | 0.03 | | 0.67 | ± | 1.09 | 0.01 |
| Post breakfast |  |  |  | |  |  |  |  |
| glycemia peak (mM) | 7.23 | ± | 0.55 | | 6.41 | ± | 0.63 | 0.05 |
| Post FlexMeal |  |  |  | |  |  |  |  |
| glycemia iAUC (mM.min) | 209.41 | ± | 56.87 | | 63.65 | ± | 23.2 | 0.01 |
| glycemia peak (mM) | 8.11 | ± | 0.87 | | 7.14 | ± | 0.61 | 0.04 |

**Table S2**. Subgroup analysis: cardiometabolic profile at baseline

Cardiometabolic profile differences at baseline between the two subgroups according to lactulose hydrogen breath test responses

Data are expressed as mean ± SD. Wilcoxon signed-rank tests were performed and considered as significant if p < 0.05.

|  | **Chitin-glucan** | | **Control** | |
| --- | --- | --- | --- | --- |
| **RAW MATERIAL** | **mg/dose** | **%** | **mg/dose** | **%** |
| Kiotransine ® (chitin-glucan from Aspergillus niger) | 1500.000 | 50.000 | _ | _ |
| maltodextrin | 1026.700 | 34.223 | 2526.700 | 84.223 |
| acidity regulator: citric acid | 300.000 | 10.000 | 300.000 | 10.000 |
| flavour | 100.000 | 3.333 | 100.000 | 3.333 |
| flavour | 50.000 | 1.667 | 50.000 | 1.667 |
| colour: beta carotene | 10.000 | 0.333 | 10.000 | 0.333 |
| anticaking agenht: silicon dioxide | 7.000 | 0.233 | 7.000 | 0.233 |
| sweetener: sucralose | 2.800 | 0.093 | 2.800 | 0.093 |
| sweetener: acesulfame K | 1.750 | 0.058 | 1.750 | 0.058 |
| sweetener: sodium saccharin | 1.750 | 0.058 | 1.750 | 0.058 |
| TOTAL | 3000.000 | 1000.000 | 3000.000 | 100.000 |

**Table S3**. Study products composition

Raw material is indicated for the 3g sachet of product
